# HIV Tat/P-TEFb Interaction: A Potential Target for Novel Anti-HIV Therapies

**DOI:** 10.3390/molecules23040933

**Published:** 2018-04-17

**Authors:** Kaori Asamitsu, Koh Fujinaga, Takashi Okamoto

**Affiliations:** 1 Department of Molecular and Cellular Biology, Nagoya City University Graduate School of Medical Sciences, Nagoya 467-8601, Japan; tokamoto@med.nagoya-cu.ac.jp; 2 Department of Medicine, Microbiology and Immunology, University of California, San Francisco, CA 94143-0703, USA

**Keywords:** HIV transcription, P-TEFb, cyclin T1, CDK9, Tat, HEXIM1

## Abstract

Transcription is a crucial step in the life cycle of the human immunodeficiency virus type 1 (HIV 1) and is primarily involved in the maintenance of viral latency. Both viral and cellular transcription factors, including transcriptional activators, suppressor proteins and epigenetic factors, are involved in HIV transcription from the proviral DNA integrated within the host cell genome. Among them, the virus-encoded transcriptional activator Tat is the master regulator of HIV transcription. Interestingly, unlike other known transcriptional activators, Tat primarily activates transcriptional elongation and initiation by interacting with the cellular positive transcriptional elongation factor b (P-TEFb). In this review, we describe the molecular mechanism underlying how Tat activates viral transcription through interaction with P-TEFb. We propose a novel therapeutic strategy against HIV replication through blocking Tat action.

## 1. Introduction

Acquired immunodeficiency syndrome (AIDS) is the number one killer among infectious diseases, with over one million victims in the world [1]. Although the prevalence of HIV infection worldwide has been reduced, the number of AIDS deaths continues to rise in developing counties. The major targets of anti-HIV-1 drugs include viral protease, reverse transcriptase, integrase, and viral attachment; none of which, however, are the rate-determining step of viral replication. Since viral reverse transcription is coupled with RNase H activity and the viral genomic RNA molecule is rapidly degraded by RNase H, the actions of reverse transcriptase and protease do not confer the rate-determining step of viral replication. Instead, it is the step of transcription from the HIV provirus by which the virus amplifies its genetic information. HIV contains the most efficient transcriptional activator called Tat. Moreover, due to the high rate of mutation during the accelerated viral transcription, which is error-prone, followed by reverse transcription, causing small deletions and insertions, the emergence of viral quasi-species is inevitable. Thus, the virus rapidly acquires the resistance to host immune responses and various anti-HIV drugs [2]. Therefore, understanding the molecular mechanism of Tat action, that endows HIV with the extraordinary transcriptional efficacy, should facilitate the development of novel therapies against this most formidable microbe.

## 2. Significance of Transcriptional Regulation of HIV Gene Expression in Viral Life Cycle

**Figure 1:**
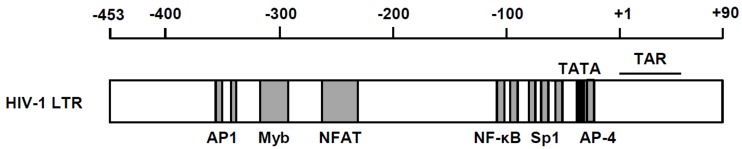
Cis-acting regulatory elements in the HIV-1 LTR. Binding sites for AP-1 (nucleotide positions: −350 to −343 and −336 to −330), Myb (−314 to −293), NFAT (−274 to −218), NF-κB (−105 to −96 and −91 to −82), SP-1 (−78 to −69, −67 to −58 and −56 to −47) and AP 4 (−22 to −17) are indicated as gray boxes. Positions of the TATA box (−27 to −24) and TAR region (+1 to +59) are shown as a black box and black line, respectively.

Besides being the rate-determining step of viral replication, HIV transcription is also the key step at which the virus maintains its latency. Therefore, we will dissect this process in two distinctive aspects: (1) positive regulation, and (2) negative regulation. There are a number of positive transcriptional regulators of host cells, including nuclear factor κB (NF-κB), Sp1 and NFAT-1, whereas negative transcription factors, including YY1 and AP-4, have been shown to be located within the viral long terminal repeat (LTR) [3,4,5,6,7,8] (Figure 1). In addition, many reports have deciphered the presence of epigenetic control in silencing the transcriptional competence of the integrated provirus [9,10,11]. Because of such promoter context, HIV replication is coordinated in accordance with the extracellular environments. For instance, extracellular signals, including immune-inflammatory signals and oxidative stress, elicit activation of inducible host transcription factor, particularly NF-κB [11]. Moreover, epigenetic stimulators such as butyric acid [12] that inhibit histone deacetylase (HDAC) could induce transcription from proviral DNA.

In addition to the transcriptional control by host cellular transcription factors, HIV encodes its own transactivator Tat [13,14]. Tat specifically binds to the HIV nascent mRNA through its interaction with the transactivation response (TAR) element [15,16], a stem-bulge-loop structure present at the very 5′ end of all viral transcripts [13]. Tat stimulates transcription in a virus-specific fashion, primarily at the step of transcriptional elongation [17]. As expected, the genetically modified HIV devoid of Tat gene loses its transcriptional competence and the ability to efficiently replicate in cells [18]. This implicates Tat as an ideal molecular target for HIV replication. In fact, a Tat inhibitor, didehydro-Cortistain A (dCA), inhibits viral rebound after treatment interruption, as demonstrated in an in vivo model of HIV infection, thus supporting the rationale for the inclusion of specific HIV transcriptional inhibitors in eradication strategies [19,20].

## 3. Tat Functional Domains and Its Binging Partners

**Figure 2:**
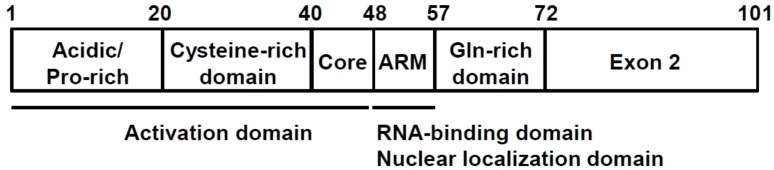
Tat functional domains.

Tat polypeptide is a 15 kDa protein and is located predominantly in the nucleus of HIV-1-infected cells. Tat is encoded by two exons and contains several critical functional regions (Figure 2) and the neighboring Arg-rich motif (known as “ARM”, spanning 49–57 amino acid residues). The ARM is essential for binding to TAR RNA, nuclear localization, and Tat protein stability [17]. The characteristic Cys-rich domain containing six Cys and one His residues is critical for zinc binding and indispensable for Tat-mediated transcription [16]. There are two additional regions of Tat, the Gln-rich domain, which is well conserved in various HIV-1 isolates, and the “exon 2” domain, which has no known definitive function [17].

The Tat actions include (1) initiation of viral transcription, (2) transcriptional elongation, and (3) stabilization of viral mRNAs. These actions appear to be mediated through interacting with distinct partner proteins. It is noted that Tat is a typical naturally denatured protein, and its 3-D structure is not established until it binds to the specific interacting molecule [21]. These features make Tat a versatile protein by making multiple interactions with host proteins to execute various biological actions, thus benefiting HIV-1 with respect to its propagation and causing AIDS. Recently, Jean et al. performed a parallel analysis of in vitro translated open reading frames (OPFs) (PLATO) approach to identify Tat binding proteins [22]. As expected, almost all these proteins are involved in transcription, indicating that the main function of Tat is still in transcription. Similar results have been reported by other studies [23,24,25].

The primary action of Tat is believed to be at the step of transcriptional elongation, where Tat interacts with cellular positive transcriptional elongation factor b (P-TEFb), which contains cyclin T1 (CycT1) and CDK9 [26,27]. In order to develop efficient anti-HIV compounds with Tat as the target, an understanding of its structure, as well as its specific molecular action, is crucial. Since Tat requires P-TEFb to stimulate HIV transcription elongation, Tat/P-TEFb interaction is an attainable target for developing new anti-HIV drugs. In addition to P-TEFb, several reports have shown that Tat interacts with importin α/β and other transcriptional regulators, including protein kinase PKR, Sp1, and the transcriptional coactivators CBP/p300 through the Tat exon 1 region [28,29,30,31,32,33,34]. Moreover, a cellular factor Tat-SF1 was identified as a cofactor for Tat-dependent transactivation of HIV transcription [35]. Tat-SF1 is an RNA binding protein that functions as a transcription elongation factor and also in splicing; thus, Tat specifically controls splicing [36,37,38]. However, the roles of those Tat-binding proteins in HIV transcription are yet to be determined. It is yet to be determined whether those proteins are promising targets for anti-HIV therapies.

Furthermore, a previous report demonstrated that Tat recruits a family of closely related multisubunit complexes called the “super elongation complexes” (SECs), rather than the isolated P-TEFb [24,25]. Thus, efficient HIV transactivation by Tat depends on the presence of complete SEC in addition to the recruitment of P-TEFb. Molecular details will be discussed in the next section.

## 4. P-TEFb as a Crucial Cofactor of Tat for Its Action

P-TEFb was initially isolated biochemically as a kinase that phosphorylates the C-terminal domain (CTD) of the large subunit of RNA polymerase II (RNAPII) [39]. The catalytic subunit of P-TEFb was first identified as PITALRE [40]. The PITALRE kinase was effectively inhibited by the ATP analog 5,6-dichlorobenzimidazole 1-β-D-ribofuranoside (DRB) [39,41], and thus is considered essential for RNAPII transcription.

Since binding between Tat and TAR was not sufficient for Tat action, additional cellular factor(s) were explored. For instance, Tat was not active in rodent cells and its activity was restored by a factor encoded in human chromosome 12, indicating the presence of a critical cofactor in this chromosome [42,43]. Rice and colleagues reported that Tat associates cellular protein kinases that phosphorylate RNAPII CTD, and was named Tat-associated kinase (TAK) [44]. Subsequently, Mancebo et al. [45] identified the Cdk9 subunit of P-TEFb as TAK, by demonstrating that Tat-dependent transcription could be blocked only by Cdk9-specific inhibitors. Furthermore, Jones and colleagues identified the cyclin subunit of P-TEFb, named Cyclin T1 (CycT1) [27]. CycT1, which is encoded in chromosome 12, interacts with Tat and TAR, and stimulates HIV transcription in non-permissive rodent cell line, meeting all the criteria of the cellular Tat co-factor [27]. Assembly of the Tat-TAR-P-TEFb complex at the HIV promoter induces the synthesis of full-length HIV viral mRNA by stimulating the CDK9-mediated hyperphosphorylation of the RNAPII CTD [45,46].

Affinity purification was performed to reveal other factors involved in the Tat-mediated transactivation and identified the transcription factors/cofactors including ELL2, AFF4, ENL, and AF9 [24,25]. These factors are now referred to as SEC. Among the SEC members, the AFF4 binding specifically induces the affinity of SEC to TAR through its interaction with CycT1 and Tat [47]. However, the precise role of the SEC complex in Tat-transactivation is yet to be determined [48]. Thus, SEC is likely involved positively in HIV transcription via P-TEFb/Tat/TAR/SEC interaction.

## 5. P-TEFb as a Positive Regulator of Transcription Elongation and the Involvement of Brd4

Functional activation of P-TEFb appears to precede the gene expression. Immediately following transcriptional initiation, RNAPII becomes trapped in the promoter proximal paused positions on most human genes, an effect known as “promoter proximal pausing (PPP)” [49,50]. In this concept, the commencement of transcription of most genes is inhibited by DRB-sensitivity inducing factor (DSIF) and negative elongation factor (NELF) [51,52] acting as a transcriptional checkpoint. This observation was initially made by in vivo analyses of the heat-shock protein genes of *Drosophila melanogaster* [53,54]. These studies revealed the short (20–60 nucleotides) nascent mRNA in the vicinity of the transcriptionally committed RNAPII just downstream of the heat-shock protein promoter. Similarly, mammalian genes including human *c-myc* and *c-fos* genes were associated with the engaged RNAPII molecules immediately downstream of transcription start site [55,56,57]. Recent genome-wide chromatin immunoprecipitation (ChIP) assays or the ChIP followed by high-throughput sequencing (ChIP-seq) have demonstrated that most RNAPII are found in the promoter-proximal regions, thus supporting the biological relevance of the PPP concept. Interestingly, RNAPII was also accumulated on the HIV promoter [58,59]. However, the accumulated nascent HIV transcripts of 59 nucleotides-long form a typical stem-bulge-loop secondary structure that is specifically recognized by the Tat [13,60]. P-TEFb phosphorylates DSIF and NELF, as well as the RNAPII CTD [39], leading to transition from “transcriptional initiation” to “productive elongation” to commence the synthesis of entire viral mRNAs. Treatment of cells with P-TEFb inhibitors DRB or flavopiridol leads to the efficient suppression of viral mRNA synthesis [61,62,63].

A recent report by Lu et al. [64] has revealed the involvement of another positive regulator, bromodomain-containing protein Brd4, together with SEC in releasing the paused Pol II. In addition, these proteins are cooperatively regulated by P-TEFbs. Brd4 binds to P-TEFb and brings it to the transcription elongation machinery via the H3K4Me mark [65,66]. In HIV transcription, Brd4 seems to regulate Tat-independent transcription (initial round of HIV transcription when Tat is not produced) [67,68]. Importantly, JQ1, which release Brd4 from chromatin, acts as a potent latency reversing agent (LRA). However, JQ1 induces P-TEFb activity [69], and it is still to be determined whether releasing Brd4 from chromatin is a necessary step of activating HIV transcription from latency.

## 6. Negative Regulation of P-TEFb by HEXIM1 and 7SK Small Nuclear Ribonucleoprotein (7SK snRNP)

**Figure 3:**
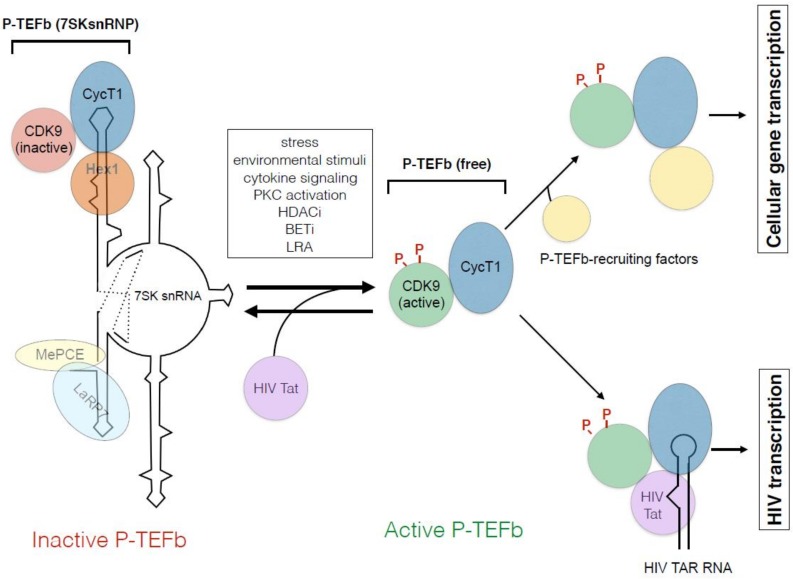
P-TEFb regulatory mechanism. In cells, most P-TEFb molecules are incorporated into 7SK snRNP, which contains 7SK snRNA, HEXIM1, MePCE, and LARP7. In 7SK snRNP, the CycT1 subunit directly binds to the central loop of 7SK snRNA and HEXIM1, which inhibits the kinase activity of Cdk9. Various stimuli, including stress, environmental stimuli, cytokine signaling, PKC activation, and treatment of cells with HDACis, BETis, and other LRAs, release P-TEFb and stimulate Cdk9 kinase activities accompanied by phosphprylation of critical T-loop (Thr 186) and central serine (Ser 175) residues. Released (free) P-TEFb can subsequently be recruited to RNAPII early elongation complex paused at promoter proximal regions of many cellular genes by various factors such as transcription factor, Brd4, Super Elongation Complex, Mediator complex, etc. HIV Tat protein can directly recruit P-TEFb to RNAPII on HIV LTR via binding with viral TAR RNA, since it can complete with HEXIM1/7SK snRNA for P-TEFb.

Several modes of regulation for the P-TEFb function are noted in cells. First, similar to cyclin molecules involved in the cell cycle control, the levels of CycT proteins are tightly regulated post-transcriptionally by micro (mi)RNAs and other mechanisms [70,71]. As a consequence, the protein level of functional P-TEFb is vanishingly low in quiescent cells [70,71], which is a major cause of HIV latency in resting CD4+T cells. P-TEFb levels in resting T-cells are significantly up-regulated by T-cell receptor (TCR) signaling and the subsequent activation of protein kinase C (PKC) [70,71]. Second, most P-TEFb molecules in cells are incorporated in 7SK small nuclear ribonucleoproteins (snRNPs) as an enzymatically inactive complex (Figure 3). The core components necessary to form 7SK snRNP are P-TEFb, HEXIM1, LaRP7, MEPCE, and 7SK snRNA [72,73,74]. Among these molecules, 7SK snRNA and HEXIM1 inhibit CDK9’s kinase activity by direct interaction, whereas LaRP7 and MEPCE are required to maintain the structure of 7SK snRNP [75,76]. Various environmental stimuli, including UV, DNA damage, oxidative stress, inhibition of HDAC and bromodomain and extra-terminal (BET) proteins, signaling cascade (PKC, MAPK, T-cell receptor signaling, etc.) and developmental cues release P-TEFb from 7SK snRNP, and activate the kinase activity of CDK9 [75,76] (Figure 3). Therefore, release of P-TEFb from 7SK snRNP represents a main regulatory step of HIV reactivation from latency. Many LRAs activate HIV transcription via this mechanism [77,78,79]. Interestingly, HIV Tat competes with HEXIM1/7SK snRNAs for binding to P-TEFb and dissociates P-TEFb from the 7SK snRNP complex [80,81]. This is partly because the 5′ stem-loop structure (SL1) of 7SK snRNA mimics that of HIV TAR RNA. Namely, the central loop of both RNA binds to CycT1, and the bulge region of 7SK snRNA binds to HEXIM1, whereas the bulge region of TAR binds to Tat [80,81,82] (Figure 3).

## 7. 3D Structure of Tat-P-TEFb Complex

P-TEFb needs to interact with various proteins in commencing the transcriptional elongation. This is most characteristically demonstrated in the Tat-mediated transactivation of HIV-1 [83]. Tat/P-TEFb 3D structure (PDB ID: 3MI9) revealed multiple hydrogen bonds forming among these component proteins upon Tat binding [84]. In addition, the crystal structure of the Tat/P-TEFb/AFF4 complex indicated that AFF4 is a scaffold protein [47,85]. Although the CycT1 TRM residues (253–260 amino acids) are disordered in the Tat/P-TEFb crystal structure, the presence of AFF4 in this complex resulted in the formation of a defined structure, suggesting the structural stabilization of this complex, leading to the transcriptional activation of HIV [47]. Thus, Tat and its interaction with P-TEFb exhibit a good example of bioactive protein complex with multiple naturally disordered protein segments that come together to form an active structure to perform a definitive biological function.

## 8. Identification of Critical Regions for Tat Transcriptional Activity within the Tat/P-TEFb Complex by In Vitro Study

The functional relationships within the Tat/TAR/P-TEFb complex and Tat transcriptional activity have been explored by many investigators. CycT1 appears to be the crucial determinant for Tat-mediated transcriptional activation. CycT1 consists of 726 residues containing a cyclin box domain (from positions 31 to 250 amino acids), which are sufficient to bind Tat and TAR to mediate the Tat action [86]. A central region of CycT1 (250–272 amino acids), termed the Tat–TAR recognition motif (TRM), is crucial for forming the Tat–CycT1–TAR ternary complex [86]. In the CycT1 TRM, the Cys residue at position 261 plays an essential role in its binding to Tat and TAR by forming a Zn^2+^-dependent interaction with other Cys and His residues within Tat at positions 1–48 [86,87,88,89]. In addition to TRM, the N-terminal cyclin box of CycT1 is also essential for Tat-mediated transactivation by binding to CDK9, Tat, or unidentified mechanisms [27,90,91,92,93].

CycT1 also contains crucial amino acids for the binding to other proteins such as HEXIM1 and AFF4 [94,95]. For instance, the Val107Glu substitution in CycT1 abolished the binding to HEXIM1 and CDK9 as well as to TAR, and the transcriptional activity of HIV was significantly down-modulated [94]. These findings demonstrate the presence of crucial amino acid residues of CycT1 molecule for determining the transcriptional competency of P-TEFb.

## 9. Identification of Critical Regions for Tat Transcriptional Activity within the Tat/P-TEFb Complex by Molecular Dynamics (MD) Simulation

MD simulation has been adopted to analyze the dynamic structural changes of protein complexes in many biological systems. For example, the dynamic movement of viral protease dimer has been deciphered by MD simulation, which exhibited spontaneous opening and reclosing of the protease flap [96].

In order to analyze the dynamic characteristics of Tat/P-TEFb interaction, MD simulations were conducted [96,97,98]. We detected an extensive hydrogen-bond network involving H1, H1′, and H2 helices of CycT1 and dynamic structural changes of the CycT1 H2′ helix upon Tat binding. These results indicate that the local structural changes of CycT1 upon binding to Tat is crucial for the Tat action [83].

Moreover, our recent MD simulation of Tat-/P-TEFb complex deciphered the hidden structure within CDK9 molecule [99]. In this study, we demonstrated the formation of a novel internal cavity within the CDK9 protein, which links the CDK9 substrate binding pocket and the enzyme catalytic center. This pocket is considered to be a suitable target for the development of specific CDK9 inhibitors.

## 10. Experimental Approaches for Studying PPI between Tat and P-TEFb in Cells

As we have discussed above, recent studies have revealed that transactivation of HIV transcription is regulated by protein-protein interaction of the Tat/P-TEFb complex. These results are helpful for identification of the lead compounds or drug candidates for new HIV inhibitors. Further drug development using high-throughput screening systems based on the chemical structure of such seed compounds should culminate in the successful development of novel HIV drugs. Because of the presence of the naturally denatured region within both Tat and CycT1, in vitro assay systems such as surface plasmon resonance assay might be difficult. Therefore, a cell-based assay for PPI utilizing modified fluorescent proteins should also be considered. These include Förster resonance energy transfer (FRET), biomolecular fluorescence complementation (BiFC), and fluorescent-based technology detecting protein-protein interactions (Fluoppi) [100,101,102,103,104,105].

FRET has been used to examine the direct interaction of proteins labeled with optically matched fluorophores [100]. The direct interaction and specific subnuclear localization between CycT1 and Tat in cells was demonstrated by this assay [101]. Since FRET has been used to examine the direct interaction of proteins labeled with optically matched fluorophores, issues of dimensional structural obstacles must be considered.

BiFC assay detects the positive interaction as fluorescent dots termed BiFC signal by using complementary fragments of fluorescent proteins such as the N-terminal and the C-terminal regions of the split YFP protein [102]. We demonstrated the recruitment of P-TEFb to the C-terminal domain of RNAPII after its release form the 7SK snRNP using this system [103]. Using this system, called “Visualisation of P-TEFb activation in Cells (V-PAC)” assay, we identified a new P-TEFb-releasing agent, 5′-azacytidine, which activates HIV transcription by releasing HEXIM-1 form P-TEFb.

Fluoppi detects PPI as fluorescent spots (“foci”) generated by concatenated multimerized proteins containing the Azami Green (AG) fluorescent protein. AG foci are formed by interactions between the assembly helper (Ash) protein and the AG-tagged protein [104]. Asamitsu et al. succeeded in the quantitative measurement of the molecular interactions among Tat, CycT1 and CDK9 and demonstrated that any third molecule enhances the binding between the other two molecules [105]. This was the first report to stabilize the overall Tat/P-TEFb complex by forming protein-protein interaction among each component of this complex in live cells.

## 11. Development of Specific Therapy

### 11.1. “Shock and Kill” or “Block and Lock”?

Although HAART significantly reduces plasma viral load to undetectable levels, latent HIV reservoirs containing the fully replication-competent virus persist throughout the body during the therapy [106,107]. Immediate cessation of the treatment results in plasma viremia (“viral rebound”), leading to AIDS. Therefore, the persistence of these latent reservoirs during HAART is the biggest obstacle to achieving an HIV cure.

As we have discussed above, transcriptional regulation from the latent HIV provirus in the infected cell plays a major role in the maintenance and the breakdown of viral latency. Thus, the Tat-dependent regulation of viral transcription has been considered a feasible target for anti-HIV therapy. This includes Tat inhibitors screened by high-throughput screening, such as Ro 5-3335 [108], TAR decoy [109] and chimeric peptide [91]. Later studies revealed repressor proteins, such as AP-4 [8] and epigenetic regulators [110]. Among these therapeutic attempts, two recent approaches, with “shock and kill” and “block and lock” concepts, are noteworthy and are currently being investigated for eliminating the latent viral reservoirs.

In the “shock and kill” strategy, the LRAs are utilized to reactivate the latently infected “silent” HIV, thus exposing HIV antigens to the immune system for immune clearance, while the combination of conventional anti-HIV reagents prevents new infection [111,112]. However, despite extensive efforts, this “shock and kill” has so far produced somewhat disappointing results, mainly because desired levels of both “shock” (reactivating HIV at a very high level from all latent reservoirs) and “kill” (efficiently eliminating HIV expressing cells by immune response and/or viral cytotoxicity) steps cannot be achieved by current regimens [106,113,114]. On the other hand, the “block and lock” strategy is to keep the latent HIV in a dormant state or drive latently infected cells into even “deeper” latency (“block and lock”) [112]. Further updates of these interesting therapeutic strategies are described below.

### 11.2. “Shock and Kill” Approach

To achieve an effective “shock and kill” approach, it is critical to achieve a high level of HIV gene expression in latently infected cells by fully reactivating HIV transcription in the absence of Tat. When Tat is produced, it binds to P-TEFb even in the presence of 7SK snRNP and recruits/activates P-TEFb for HIV transcription [115]. Although several molecular pathways are involved in maintaining HIV latency, a common pathway necessary to be induced is the P-TEFb pathway, which must be achieved in two steps; induction of CycT1 protein level and stimulating Cdk9 kinase activity by releasing P-TEFb from 7SK snRNP (Figure 3) [79]. All “shock and kill” strategies must have these two activities. Currently, there are several classes of LRAs, including HDACis, BETis, PKC agonists, Toll-like receptor agonists, and other unclassified compounds, such as disulfram [79,106,111,116,117], being tested in pre-clinical or clinical settings. Among these, HDACis, BETis, PKC agonists and disulfram all have strong activities of releasing P-TEFb from 7SK snRNP [79] (Figure 3). Therefore, LRAs do not seem to affect P-TEFb/Tat interaction. Moreover, these LRAs do not increase the level of CycT1 proteins, and therefore, these LRAs alone do not activate HIV transcription from HIV latently infected resting T cells in which CycT1 proteins are diminished. On the other hand, PKC agonists can increase CycT1 protein levels in resting CD4+T cells [79].

Therefore, an effective “shock and kill” can be achieved only by a combination of a PKC agonist and additional LRAs, which releases P-TEFb from 7SK snRNP. Further information about current clinical trials using LRAs can be found in [117,118]. The “kill” part is equally important in achieving an HIV cure by “shock and kill”. However, this is beyond the scope of this review, and more details can be found elsewhere [106,111,119,120].

### 11.3. Use of Natural Products for “Shock and Kill” Therapy

Most PKC agonists used in in vitro experiments are not very promising agents in preclinical or clinical settings, since they are too toxic (PMA) or too expensive to synthesize (Bryotatin A, Prostratin, etc.). However, ingenol family compounds are very potent PKC agonists with low toxicity and low production costs, representing potential candidates for clinical trials [121,122]. In addition, some ingenols have already been approved for clinical use (for non-HIV diseases). Interestingly, euphorbia plants contain high levels of ingenol B. In particular, euphorbia kansui has been used in Chinese traditional medicine for thousands of years. Therefore, we tested whether extract of euphorbia kansui (kansui) had the ability to reactivate HIV transcription from latently infected cells singly and/or in combination with other LRAs. Kansui activates HIV expression in various HIV latency models, including PBMCs from HAART-treated HIV+ patients. Importantly, kansui exhibits a strong synergy with HDACis or BETis, indicating that kansui can be used in combination with these LRAs to achieve a high level of HIV expression in latently infected cells [78]. Finally, using natural products such as kansui would greatly reduce the cost of treatment, which would contribute to providing affordable options to the patients in areas where expensive Western medicines cannot be accessible [123].

### 11.4. “Block and Lock” Approach and Tat/TAR/P-TEFb Interaction

While Tat/TAR/P-TEFb interaction might not be a reasonable target for efficient “shock and kill” strategy, it can be a promising target for “block and lock” strategies. In fact, “TAR decoys”, sequestering Tat and P-TEFb, inhibit HIV transcription [109]. Similarly, using peptides or chimeric proteins that recruit the catalytically inactive P-TEFb complex to TAR could efficiently block Tat-dependent transcription [91]. Recently, Valente and colleagues reported that dCsA exhibited potent activity for interfering with the Tat/TAR interaction to block HIV replication and promoting viral latency [124,125]. Cells treated with dCsA exhibited a significant delay in HIV reactivation upon LRA treatment, indicating that dCsA brings the latently HIV-infected cells into even deeper latency [20]. Molecular studies suggested that dCsA binds the ARM of Tat and disrupts Tat/TAR interaction [20]. Although additional structure-function studies are required for the precise molecular action of dCsA, these studies have provided a fascinating hypothesis that targeting the Tat-TAR axis should be a promising approach to achieve effective “block and lock” treatment in HIV-positive patients.

## 12. Conclusions

Since viral transcription is considered to be the rate-determining step of viral replication, a number of research groups have been pursuing the clarification of the specific viral transcriptional mechanism for the development of novel therapy. The discovery of Tat-mediated transcriptional activation has led to the biochemical clarification of the Tat/TAR interaction and identified the involvement of host transcriptional elongation factors such as P-TEFb and SEC as positive regulators and HEXM1 plus 7SK snRNP as negative regulators. Moreover, a number of studies using structural biology and structural bioinformatics have deciphered the dynamism of macromolecular interactions or the atomic details of the PPIs that are crucially involved in Tat action.

Considering the PPI as the physico-chemical basis for biomolecular interactions, resolution of mechanistic dynamism should be a prerequisite for the understanding of biological actions and development of small molecular compounds that inhibit such specific PPI. Similarly, important issues in such scientific explorations include the use of more relevant assay systems, ideally using live cells (such as Fluoppi), and the availability of chemical libraries with extensive structural variations, including a wide variety of bioavailable natural compounds. Identification of the PPI surface of the molecular complex with biological relevance and the “pharmacophore” or “drug-binding cavity” should greatly facilitate drug development. The small molecular compounds thus identified should interfere with the specific PPI, thus selectively inhibiting its biological actions. Such compounds could be used to efficiently block viral replication by inhibiting the viral transcription, the only rate-determining step for amplifying viral genomic information. Such therapeutic strategy could be applied to conventional anti-HIV therapy and even prevent the emergence of drug-resistant viral clones. Systemic approaches may provide a foundation for the development of “rational” drugs based on the molecular understanding of the disease.

## Abbreviations

The following abbreviations are used in this manuscript:

CycT1	cyclin T1
HIV	Human immunodeficiency virus type 1
LRA	latency reversing agents
P-TEFb	positive transcriptional elongation factor b
RNAPII	RNA polymerase II
TAR	transactivation response
